# Cardiovascular imaging in children and adults following Kawasaki disease

**DOI:** 10.1007/s13244-015-0422-0

**Published:** 2015-07-27

**Authors:** S. M. Dietz, C. E. Tacke, I. M. Kuipers, A. Wiegman, R. J. de Winter, J. C. Burns, J. B. Gordon, M. Groenink, T. W. Kuijpers

**Affiliations:** Department of Paediatric Haematology, Immunology and Infectious diseases, Emma Children’s Hospital, Academic Medical Center (AMC), Meibergdreef 9, 1105 AZ Amsterdam, The Netherlands; Department of Paediatric Cardiology, Emma Children’s Hospital, AMC, Amsterdam, The Netherlands; Department of Paediatric Metabolic Diseases, Emma Children’s Hospital, AMC, Amsterdam, The Netherlands; Department of Cardiology, AMC, Amsterdam, The Netherlands; Department of Paediatrics, University of California San Diego and Rady Children’s Hospital, San Diego, CA USA; Department of Interventional Cardiology, San Diego Cardiac Centre and Sharp Memorial Hospital, San Diego, CA USA; Department of Radiology, AMC, Amsterdam, The Netherlands

**Keywords:** Kawasaki disease, (Cardiac) MRI, MDCT, Coronary aneurysm, Cardiac imaging

## Abstract

Kawasaki disease (KD) is a paediatric vasculitis with coronary artery aneurysms (CAA) as its main complication. Two guidelines exist regarding the follow-up of patients after KD, by the American Heart Association and the Japanese Circulation Society. After the acute phase, CAA-negative patients are checked for cardiovascular risk assessment or with ECG and echocardiography until 5 years after the disease. In CAA-positive patients, monitoring includes myocardial perfusion imaging, conventional angiography and CT-angiography. However, the invasive nature and high radiation exposure do not reflect technical advances in cardiovascular imaging. Newer techniques, such as cardiac MRI, are mentioned but not directly implemented in the follow-up. Cardiac MRI can be performed to identify CAA, but also evaluate functional abnormalities, ischemia and previous myocardial infarction including adenosine stress-testing. Low-dose CT angiography can be implemented at a young age when MRI without anaesthesia is not feasible. CT calcium scoring with a very low radiation dose can be useful in risk stratification years after the disease. By incorporating newer imaging techniques, detection of CAA will be improved while reducing radiation burden and potential complications of invasive imaging modalities. Based on the current knowledge, a possible pathway to follow-up patients after KD is introduced.

*Key Points*

• *Kawasaki disease is a paediatric vasculitis with coronary aneurysms as major complication.*

• *Current guidelines include invasive, high-radiation modalities not reflecting new technical advances.*

• *Cardiac MRI can provide information on coronary anatomy as well as cardiac function.*

• *(Low-dose) CT-angiography and CT calcium score can also provide important information.*

• *Current guidelines for follow-up of patients with KD need to be revised.*

## Introduction

Kawasaki disease (KD) is an acute paediatric vasculitis that mainly affects children younger than 5 years of age. Although the exact cause is still unknown, it is thought to be caused by an infectious agent in genetically predisposed children. This fits with the observed epidemiology of seasonal occurrence throughout the northern hemisphere, the increased incidence in children of Japanese descent, and the established association with specific genetic polymorphisms [1, 2]. In about 25 % of untreated patients, coronary artery aneurysms (CAA) will develop during the acute phase of KD. Since the introduction of high-dose intravenous immunoglobulin as effective treatment, the percentage of CAA has dropped to 5–7 % for patients treated within 10 days after fever onset [3]. For patients not treated within 10 days, the rate of developing aneurysms may be higher [4].

During CAA formation it is thought that an influx of inflammatory cells leads to dissociation and disruption of the medial and internal elastic lamina layer. Even when the lumen of the artery returns to its normal size, the artery wall remains damaged, although the extent of the damage varies among patients. In one autopsy study, active remodelling of the artery was seen many years after the acute disease in children with aneurysms [5]. The KD-related vasculopathy during follow-up with characteristic myointimal proliferation and/or layering of thrombus can result in progressive stenosis, which may lead to ischemic cardiomyopathy.

**Fig. 1:**
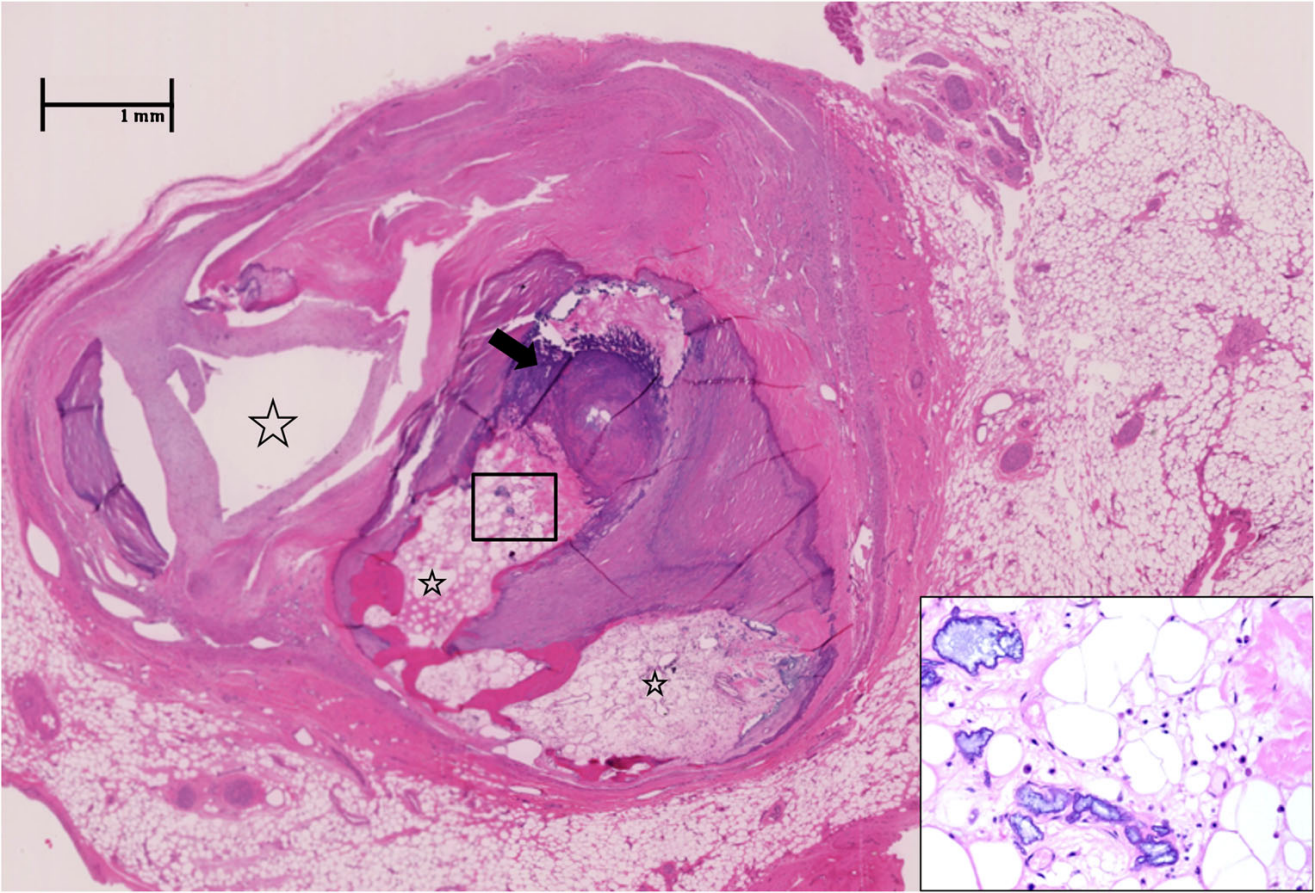
Coronary artery lesion in Kawasaki disease during follow-up. Extensive calcification (*arrow*) with ossification and bone marrow elements (insert, 400×) in the thrombosed and re-canalized left anterior descending artery from the explanted heart of a 29-year-old man who suffered from Kawasaki disease at age 3 years. The aneurysms remodelled, and the patient was discharged from follow-up at the age of 7 years when the coronary artery appeared normal by echocardiogram. The patient presented with progressive congestive symptoms at age 29 years and required cardiac transplantation. Characteristic ‘lotus root’ appearance of the artery results from thrombosis with recanalization (*stars*). Only one lumen remains patent (*far left*). Haematoxylin and eosin stain, 40×

Calcification of the damaged artery is progressive after the acute phase of the disease and may develop in coronary arteries with lesions that persist or, although rarely, in transiently dilated arteries when the vessel lumen has normalized [6]. The extensive calcification of the coronary artery wall is typical of the pathology observed in the remodelled lesional wall (Fig. 1).

CAA are diagnosed by echocardiography during the acute or subacute phase of the disease. CAA are traditionally classified according to the definitions of the Japanese Ministry of Health: a luminal diameter of >3 mm in children <5 years of age, >4 mm in children ≥5 years of age, or a diameter 1.5 times the size of an adjacent segment or an irregular lumen in children ≥5 years of age. A giant aneurysm is defined as an aneurysm with an internal diameter of ≥8 mm or a diameter >4 times that of an adjacent segment in children ≥5 years old [7, 8]. However, over the past years it has become clear that z-scores, adjusted for body-surface area, may be a better indicator of any serious enlargement [9, 10]. Using this approach, a z-score of ≥2.5 is considered an aneurysm; a z-score of ≥10 is a giant aneurysm.

It is clear that a 6 mm aneurysm in a 6-month-old child represents a more severely damaged artery than a 6 mm aneurysm in a 6-year old child. As z-scores might differ from the traditional definitions, especially in younger and smaller children, this may alter their (long-term) therapy and monitoring.

It should be emphasized that all patients who develop CAA carry a lifelong increased risk for coronary thrombosis and stenotic coronary lesions that may result in myocardial ischemia, infarction, and sudden death [11].

There has been much debate about the appropriate way to follow-up on our KD patients, both with and without CAA. Several imaging techniques have been proposed and different guidelines have been published. The aim of this paper was to analyse the existing guidelines regarding the cardiovascular follow-up of patients after KD and to propose a new pathway for follow-up, based on the current knowledge and our own experience.

### Current guidelines

Among the many issues concerning clinical management of patients with a history of KD, several controversies exist. One of these is the timing and need for long-term follow-up. Two consensus guidelines have been published, one by the American Heart Association (AHA) in 2004, and one by the Japanese Circulation Society (JCS) in 2014 [8, 12]. These guidelines are summarized in Tables 1 and 2, starting at 1 year after the acute disease. In both guidelines, patients are risk-stratified based on the severity of the coronary artery lesions and likelihood of complications, including myocardial ischemia and congestive heart failure.

**Table 1:** Summary of AHA guidelines regarding the follow-up of patients with Kawasaki disease, starting at 1 year

Risk level	Diagnostic testing	Interval	Invasive testing
No CAA	Cardiovascular risk assessment	5 years	None
Transient CAA^a^	Cardiovascular risk assessment	3–5 years	None
Small-medium CAA (>3 mm but <6 mm, z-score 3–7)	Cardiovascular risk assessment Echocardiogram + ECG	1 year 1 year	Invasive CAG if non-invasive test suggests ischemia
	Stress test with MPI	2 years	
Large CAA (≥6 mm) or Multiple or complex CAA in 1 artery	Echocardiogram + ECG	one half year	Invasive CAG after 6–12 months and if any test or clinical finding suggests ischemia
	Stress test with MPI	1 year	
Coronary artery obstruction	Echocardiogram + ECG	one half year	Invasive CAG for therapeutic options and if new onset or worsening myocardial ischemia is suggested
	Stress test with MPI	1 year	

From: Newburger et al., Circulation. 2004;110(17):2747–71^8^

*CAG* conventional angiography, *MPI* myocardial perfusion imaging

^a^Disappearing within 6–8 weeks after the onset of Kawasaki disease

**Table 2:** Summary of JCS guidelines regarding the follow-up of patients with Kawasaki disease, starting at 1 year

	Diagnostic testing	Interval	Invasive testing
No or transient CAA^a^	Exercise ECG + echocardiogram	Once, 5 years after disease ^b^	None
Small CAA (≤4 mm)^a^			
- Regressed	(Exercise) ECG + echocardiogram	Annual until age 7 Triennial until age 16	None
- Persisting	(Exercise) ECG + echocardiogram	3 months (until normalisation)	None
- Regressed or persisting	In patients ≥ 10 years after onset, consider MDCT or MRCA at final evaluation.		
Medium CAA (>4 − <8 mm)^a^			
A. CAA > 4 − <6 mm			
- Regressed	ECG + echocardiogram X-ray + exercise ECG when necessary/feasible MDCT or MRCA	Annual 5 years	Selective CAG on individual basis
- Persisting	ECG + echocardiogram X-ray + exercise ECG when necessary/feasible MDCT or MRCA	3–6 months 5 years	Selective CAG on individual basis
B. CAA 6 − <8 mm			
- Regressed	ECG + echocardiogram X-ray + exercise ECG when necessary/feasible MDCT or MRCA Appropriate combination of techniques ^c^	Annual 5 years	Invasive CAG once during convalescence and at time of disappearance of dilatation
- Persisting	ECG + echocardiogram X-ray + exercise ECG when necessary/feasible MDCT or MRCA Appropriate combination of techniques ^c^	3–6 months 5 years	Invasive CAG once during convalescence and at time of disappearance of dilatation
Giant CAA (≥8 mm)^a^	Tailor-made treatment with appropriate combination of (exercise) ECG, echocardiogram and other techniques ^c^	3–6 months	Invasive CAG during early convalescence phase

From: Group JCSJW: Circ J 2014, 78(10):2521–2562^8^

*MDCT* Multidetector CT, *MRCA* MR Coronary Angiography

^a^Measured at 30 days after the onset of KD

^b^Additional follow-up from the second to fifth year and after the fifth year can be scheduled individually through consultation between patient and physician

^c^Imaging techniques include stress echocardiography, stress myocardial perfusion scintigraphy, invasive coronary angiography (CAG), Intravenous Ultrasound, Cardiac Magnetic Resonance Imaging, Magnetic Resonance Angiography and Multidetector CT

In children without CAA or with early, transient coronary artery dilatations, the AHA advises echocardiograms at initial diagnosis and 2 and 6–8 weeks later. A repeat echocardiogram at 1 year is considered optional. Subsequently, only periodic assessment and counselling about cardiovascular risk factors are recommended. To evaluate patients without CAA, the JCS advises an ECG and echocardiogram at 1, 2, 6 and 12 months, and a final evaluation at 5 years with an exercise ECG.

For patients diagnosed with persisting CAA after the acute phase, both guidelines advise a more intensive and prolonged follow-up with additional imaging modalities.

In children with small to medium aneurysms, the AHA advises an annual routine follow-up and biennial stress testing with myocardial nuclear perfusion scans. In case of large and/or multiple or complex aneurysms within one artery, the AHA recommends an invasive coronary angiography (CAG) to be performed routinely 6–12 months after the disease onset and repeated if the non-invasive stress test or clinical signs suggest myocardial ischemia.. It should be noted that these guidelines were last revised in 2004 and many aspects of clinical care and imaging have changed during this interval.

The JCS has recently published a revised guideline in which the committee recommends follow-up for patients with small aneurysms at 30 days after the acute illness with an echocardiogram and ECG every 3 months until the dilatation has disappeared. Patients with medium-sized aneurysms should be more intensely evaluated, i.e., every 1–3 months with (exercise) ECG, echocardiography and chest X-ray until dilatation is no longer observed and should undergo MDCT or MR Coronary Angiography (MRCA) every 5 years. This scheme differs from the AHA guidelines.

For those with larger CAA, JCS advises a follow-up in a risk-stratified manner using methods that are largely overlapping the AHA recommendations. There are no specific recommendations for imaging modalities.

### Concerns

There are two main drawbacks to the current guidelines for follow-up imaging of patients with KD.

1. The invasive nature and high radiation exposure of the advised follow-up modalities do not reflect technical advances in the field of cardiovascular imaging and result in possible complications and unnecessary hazardous radiation exposure.
2. Coronary aneurysms, especially those more distal in the coronary tree, can be missed by echocardiography, potentially leading to underestimation of disease severity.

For this reason the use of alternative imaging methods should be considered, and we suggest a feasible, accurate, and sustainable follow-up scheme for KD as outlined below.

### Cardiac Magnetic Resonance Imaging (CMRI)

CMRI is a robust imaging technique that is being widely adopted by many centres for the evaluation of coronary artery disease. Besides coronary anatomy, CMRI provides clinically relevant information about myocardial ischemia, infarction, inflammation, and function. In experienced centres, cardiac nuclear scintigraphy has been largely abandoned because of low sensitivity and high radiation exposure while being outperformed by CMRI [13]. The current availability of CMRI and expertise to apply CMRI varies greatly across centres.

Using a comprehensive CMRI protocol with adenosine stress testing during the follow-up of 63 children with KD, we showed that CMRI was safe and able to identify coronary artery pathology, signs of ischemia, and previous myocardial infarction [14]. CMRI, including MRCA, compared favourably with echocardiography for the identification of CAA; in six patients CAA were missed by echocardiography and identified by CMRI. Mavrogeni et al. compared coronary MRCA to conventional CAG in 12 patients [15]. The two techniques agreed completely in the detection of aneurysms, and the maximal aneurysm diameters were similar. A larger study from Suzuki et al. compared coronary MRCA in 106 patients with echocardiography and found 28 additional aneurysms with MRCA [16]. When comparing coronary MRCA to conventional CAG in 12 patients with imaging methods <6 months apart, they found no significant difference in arterial diameter using bright blood imaging, although one occlusion of the circumflex artery was only detected by conventional CAG.

Coronary artery evaluation can be easily combined with the assessment of ischemia, infarction, ventricular dimensions, and function within the same procedure, for which CMRI has become the standard [17].

Based on our experience with CMRI in more than 150 patients with KD, a 2-step CMRI protocol was introduced in 2012. This CMRI is performed on a 1.5-T whole-body MRI scanner equipped with cardiac software. The first step of this CMRI protocol, consisting of MRCA and cardiac function evaluation, is offered routinely during adolescence, when the teenager is able to fully cooperate. An ECG-gated 3D steady-state free precession sequence with T2 and fat saturation prepulses is used to visualize the coronary arteries. If coronary abnormalities have been observed by echocardiography on two or more different occasions, the second-step of our CMRI protocol is also performed in one procedure. If CAA are observed unexpectedly upon the first step of CMRI, a second step will subsequently be performed.

The second step involves the administration of intravenous contrast (0.1 mmol/kg of gadolinium) and adenosine (140 ug/kg/min) to detect myocardial perfusion abnormalities and myocardial scar. Standard, commercially available, CMRI techniques are used with fast single shot T1-weighted gradient echo sequences (e.g., Turbo-FLASH, TFE) for perfusion imaging and segmented inversion recovery sequences for myocardial scar imaging [18, 19].

We introduced this 2-step protocol because perfusion abnormalities and ischemia upon adenosine stress were detected only in patients with persistent CAA.

Based on current data, it is unlikely that patients without CAA will omit develop clinically significant cardiovascular changes from their KD. However, in patients that are children with missed CAA by echocardiography, long-term complications can still occur.

### Low-radiation dose CT angiography

Although CMRI is a feasible technique in early adolescence, assessment of younger children with CAA often requires general anaesthesia and is, therefore, less suitable.

ECG-gated multislice CT is an attractive alternative imaging technique for the younger patient. In teenagers with a history of KD, CT-angiography confirmed all aneurysms, stenoses, and occlusions previously demonstrated by invasive CAG [20]. In another study, aneurysms were detected by CT-angiography in young children with a history of KD that had been missed by echocardiography [21].

Although effective, traditional CT-angiography carries a high radiation exposure, especially harmful for young children. Low-radiation dose CT-angiography with prospective ECG triggering is increasingly available. The use of next generation scanners decreases the radiation burden from a median of 6.9 mSv per scan down to a median of 1 mSv on a 128 dual-source CT scanner [22]. New imaging protocols can be used to reduce the radiation dose for which the use of prospective instead of retrospective ECG-triggering is most important. The use of a single heart beat (high-pitch) protocol has been shown to result in a reduction of the radiation dose requirements even further. On the other hand, studies are conflicting as to whether the image quality is reduced in patients with a relatively high heart rate, hence, in most children [22, 23]. With the use of iterative reconstruction, the image-to-noise ratio can be reduced such that lower tube currents can be used [24]. In children with congenital heart disease, this has already shown to generate qualitatively excellent results, but studies evaluating the coronary arteries in patients following acute KD have not yet been performed [25].

A recent study in infants and young children with KD showed that low-dose, free-breathing prospective ECG-triggered CT-angiography without the use of beta-blockers diagnosed all CAA detected with ultrasound *plus* the more distal aneurysms and dilatations [26]. The data acquisition window was set to 40 % of the RR-interval. The effective radiation dose was calculated at 0.32 to 0.40 mSv, depending on the patient’s age. Although this was a small study in 19 patients, it is very promising that good images could be obtained without much radiation exposure and no pre-medication. Apart from replacing invasive CAG in an early stage of follow-up, low-radiation dose CT-angiography can also be used at a later stage to check for the development of stenosis in patients with CAA.

### CT calcium scoring

Whereas CMRI is most useful for functional imaging, calcifications can be best picked up by CT imaging. CT calcium scoring, a technique without intravenous contrast, measures calcification in the coronary arteries, which was a strong predictor of coronary events in a large adult cohort of patients without evidence for cardiovascular disease at enrolment [27]. It can be used to detect coronary calcifications that may have been missed by echocardiography or CMRI and is associated with a very low radiation dose of approximately 1 mSv or less with prospective ECG triggering [6, 28].

**Fig. 2:**
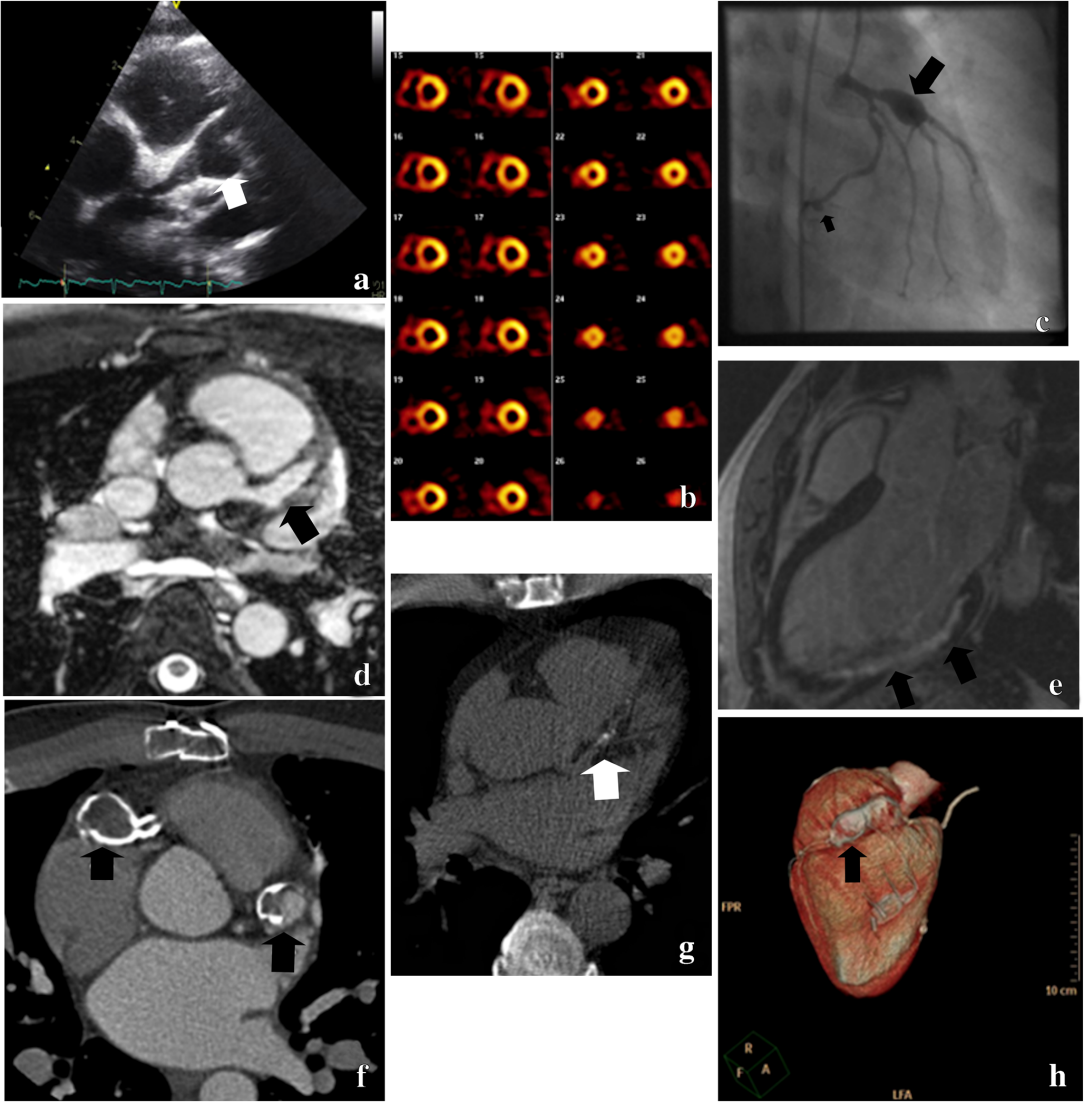
Imaging techniques for the follow-up of Kawasaki disease. **a** Echocardiogram of a giant aneurysm of the left main coronary artery (LMCA) and LAD. **b** Stress and rest SPECT Technetium-99M scan (myocardial perfusion scan) demonstrates ischemia of the inferior and septal wall. **c** Conventional CAG shows a giant aneurysm of the LAD and a smaller aneurysm of the right circumflex artery (RCX). **d** Cardiac MRI shows an aneurysm of the LAD. **e** Cardiac MRI indicates a myocardial infarction of the infero-posterior wall. **f** Multi-slice CT contrast-enhanced angiography with calcified aneurysms of the proximal LAD and right coronary artery (RCA). **g** CT calcium-score with calcifications of the proximal LAD. **h** 3D-CT angiography with a calcified aneurysm of the RCA

Although the exact clinical implication of small coronary artery calcifications is still unknown, CT calcium scoring may be useful in risk-stratification of recently identified patients whose aneurysms were missed in the past or not well monitored since the acute phase. In such patients, the aneurysm may have remodelled such that the lumen is of normal internal diameter but the arterial wall is significantly damaged and associated with calcium deposition. These patients are at risk for late stenosis and must be carefully followed. Figure 2 illustrates the different purposes of conventional techniques, CMRI and CT.

### Recommendations

**Fig. 3:**
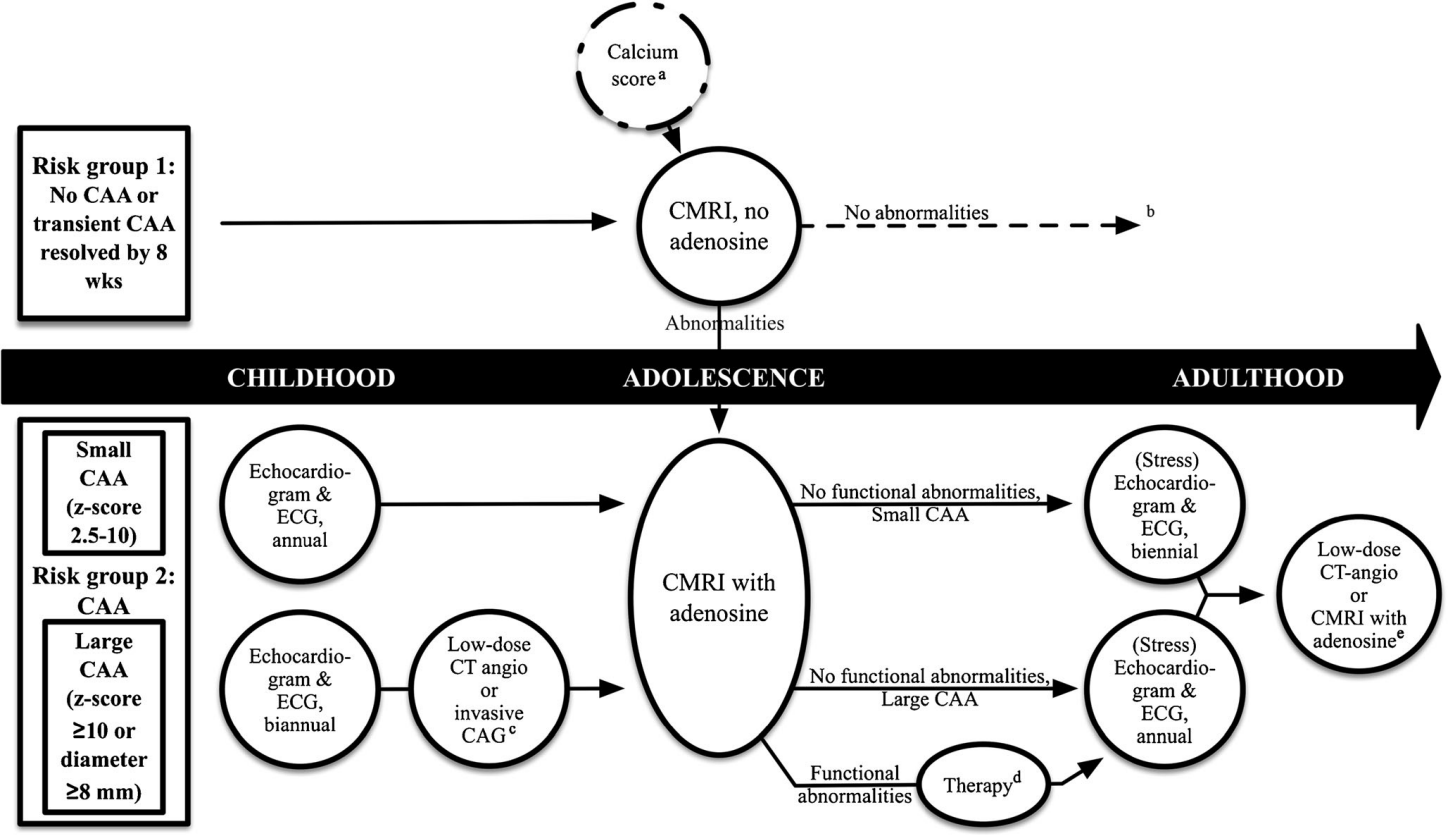
Flowchart for the monitoring of Kawasaki disease with current imaging modalities starting at 1 year after the disease. ^a^When information is lacking about coronary arterial aneurysms (CAA) status, a calcium score may be indicated as a screening method. If positive, a CMRI with adenosine should be performed. ^b^Long-term follow-up (cardiovascular counselling) of risk group 1 may be dictated by national health care policies and future studies. ^c^According to the availability and experience of a centre with (low-dose) CT angiography. ^d^Which of the different revascularization options best improves prognosis is unclear to date. ^e^Additional tests to evaluate for progression to stenotic lesions

All current guidelines are based on the opinion of experts. Evidence-based KD guidelines for cardiovascular assessment of KD patients do not exist. Based on the current knowledge and our own experience regarding different imaging modalities, we herewith suggest a pathway for follow-up in patients with KD into adulthood (Fig. 3). Advantages and disadvantages of the different techniques are outlined in Table 3.

**Table 3:** Advantages and disadvantages of imaging techniques

Imaging technique	Advantage(s)	Disadvantage(s)
Echocardiography	Non-invasive Cheap	Distal coronary arteries not visible
CAG	Complete image of coronary tree	Invasive, possible complications Need of anaesthesia Radiation exposure
CMRI	Visualisation of distal aneurysms Functional assessment	Need of anaesthesia in younger children
CT-angiography	Visualisation of distal aneurysms and stenosis Widely available	Radiation exposure
CT-calciumscore	Visualisation of calcifications late after disease Low-radiation exposure	Not suitable for aneurysm information Only applicable late after acute disease

*CAG* conventional angiography, *CMRI* Cardiac MRI

By not performing frequent echocardiography after the acute phase, costs are kept to a minimum. Furthermore, by detecting missed aneurysms or other lesions on CMRI, possible complications such as myocardial infarction can be prevented. It must be stated that this pathway will depend on the available technical quality and experience of a centre with CMRI, which varies across centres.

For patients with CAA, the pathway must be customized depending on the extent of the lesions. For instance, a young child with giant aneurysms and suspected ischemia will require invasive or CT-angiography, as well as MRI under general anaesthesia, repeated regularly as an individual approach, to obtain the best interpretation of CAA and functional stenosis.

In contrast to infants and children, adolescents and adults are able to report cardiac symptoms of ischemia. Asymptomatic adults with aneurysms must be followed for early signs of ischemia upon (stress) echocardiography, but the frequency of additional imaging studies can largely be guided by symptoms. Since echocardiography in general is insensitive for evaluating stenosis, symptomatic adults require other imaging techniques.

In addition to the techniques mentioned above, in a selected group of patients, conventional CAG can be considered when a catheter-based intervention is contemplated. Invasive CAG can be combined with fractional flow reserve to obtain hemodynamic and structural data to help guide therapeutic decisions [29]. These tests for interventional decision-making have not often been reported in KD and are beyond the scope of this article.

### Future studies

Future studies are needed to evaluate the risks and benefits of different cardiovascular imaging modalities and the most appropriate timing of such imaging to optimize care for KD patients. Evidence is lacking about the long-term effects of KD, especially in children without prior evidence of CAA. We might not appreciate late cardiovascular sequelae that may become clinically important later in life. In order to answer these questions, long-term follow-up is required.

## Conclusion

In summary, we believe that the current guidelines for follow-up of patients with KD need to be revised. By incorporating new imaging techniques in our guidelines, detection of CAA will be improved, while reducing the radiation burden and potential complications of invasive angiography. This may enable better adherence to the guidelines in clinical practice and improve follow-up of patients into adulthood.

## References

[1] Burns JC, Herzog L, Fabri O (2013). Seasonality of Kawasaki disease: a global perspective. PLoS One.

[2] Onouchi Y (2012). Genetics of Kawasaki disease: what we know and don’t know. Circ J.

[3] Newburger JW, Takahashi M, Beiser AS (1991). A single intravenous infusion of gamma globulin as compared with four infusions in the treatment of acute Kawasaki syndrome. N Engl J Med.

[4] Tacke CE, Breunis WB, Pereira RR, Breur JM, Kuipers IM, Kuijpers TW (2014). Five years of Kawasaki disease in the Netherlands: a national surveillance study. Pediatr Infect Dis J.

[5] Suzuki A, Miyagawa-Tomita S, Komatsu K (2000). Active remodeling of the coronary arterial lesions in the late phase of Kawasaki disease: immunohistochemical study. Circulation.

[6] Kahn AM, Budoff MJ, Daniels LB (2012). Calcium scoring in patients with a history of Kawasaki disease. JACC Cardiovasc Imaging.

[7] Research Committee on Kawasaki Disease (1986). Report of the Subcommittee on Standardization of Diagnostic Criteria and Reporting of Coronary Artery Lesions in Kawasaki Disease.

[8] 8 Group JCSJW (2014). Guidelines for diagnosis and management of cardiovascular sequelae in Kawasaki disease (JCS 2013). Digest version. Circ J.

[9] McCrindle BW, Li JS, Minich LL (2007). Coronary artery involvement in children with Kawasaki disease: risk factors from analysis of serial normalized measurements. Circulation.

[10] Manlhiot C, Millar K, Golding F, McCrindle BW (2010). Improved classification of coronary artery abnormalities based only on coronary artery z-scores after Kawasaki disease. Pediatr Cardiol.

[11] Kato H, Sugimura T, Akagi T (1996). Long-term consequences of Kawasaki disease. A 10- to 21-year follow-up study of 594 patients. Circulation.

[12] Newburger JW, Takahashi M, Gerber MA (2004). Diagnosis, treatment, and long-term management of Kawasaki disease: a statement for health professionals from the Committee on Rheumatic Fever, Endocarditis and Kawasaki Disease, Council on Cardiovascular Disease in the Young, American Heart Association. Circulation.

[13] Lee DC, Simonetti OP, Harris KR (2004). Magnetic resonance versus radionuclide pharmacological stress perfusion imaging for flow-limiting stenoses of varying severity. Circulation.

[14] Tacke CE, Kuipers IM, Groenink M, Spijkerboer AM, Kuijpers TW (2011). Cardiac magnetic resonance imaging for noninvasive assessment of cardiovascular disease during the follow-up of patients with Kawasaki disease. Circ Cardiovasc Imaging.

[15] Mavrogeni S, Papadopoulos G, Douskou M (2004). Magnetic resonance angiography is equivalent to X-ray coronary angiography for the evaluation of coronary arteries in Kawasaki disease. J Am Coll Cardiol.

[16] Suzuki A, Takemura A, Inaba R, Sonobe T, Tsuchiya K, Korenaga T (2006). Magnetic resonance coronary angiography to evaluate coronary arterial lesions in patients with Kawasaki disease. Cardiol Young.

[17] Tacke CE, Romeih S, Kuipers IM, Spijkerboer AM, Groenink M, Kuijpers TW (2013). Evaluation of cardiac function by magnetic resonance imaging during the follow-up of patients with Kawasaki disease. Circ Cardiovasc Imaging.

[18] Takx RA, Blomberg BA, El Aidi H et al (2015) Diagnostic accuracy of stress myocardial perfusion imaging compared to invasive coronary angiography with fractional flow reserve meta-analysis. Circ Cardiovasc Imaging 8(1) 10.1161/CIRCIMAGING.114.002666 25596143

[19] Rajiah P, Desai MY, Kwon D, Flamm SD (2013). MR imaging of myocardial infarction. Radiographics.

[20] Carbone I, Cannata D, Algeri E (2011). Adolescent Kawasaki disease: usefulness of 64-slice CT coronary angiography for follow-up investigation. Pediatr Radiol.

[21] Xing Y, Wang H, Yu X, Chen R, Hou Y (2009). Assessment of coronary artery lesions in children with Kawasaki disease: evaluation of MSCT in comparison with 2-D echocardiography. Pediatr Radiol.

[22] Ghoshhajra BB, Lee AM, Engel LC (2014). Radiation dose reduction in pediatric cardiac computed tomography: experience from a tertiary medical center. Pediatr Cardiol.

[23] Neefjes LA, Dharampal AS, Rossi A (2011). Image quality and radiation exposure using different low-dose scan protocols in dual-source CT coronary angiography: randomized study. Radiology.

[24] Naoum C, Blanke P, Leipsic J (2015). Iterative reconstruction in cardiac CT. J Cardiovasc Comput Tomogr.

[25] Zheng M, Zhao H, Xu J, Wu Y, Li J (2013). Image quality of ultra-low-dose dual-source CT angiography using high-pitch spiral acquisition and iterative reconstruction in young children with congenital heart disease. J Cardiovasc Comput Tomogr.

[26] Duan Y, Wang X, Cheng Z, Wu D, Wu L (2012). Application of prospective ECG-triggered dual-source CT coronary angiography for infants and children with coronary artery aneurysms due to Kawasaki disease. Br J Radiol.

[27] Detrano R, Guerci AD, Carr JJ (2008). Coronary calcium as a predictor of coronary events in four racial or ethnic groups. N Engl J Med.

[28] Gerber TC, Carr JJ, Arai AE (2009). Ionizing radiation in cardiac imaging: a science advisory from the American Heart Association Committee on Cardiac Imaging of the Council on Clinical Cardiology and Committee on Cardiovascular Imaging and Intervention of the Council on Cardiovascular Radiology and Intervention. Circulation.

[29] De Bruyne B, Pijls NH, Kalesan B (2012). Fractional flow reserve-guided PCI versus medical therapy in stable coronary disease. N Engl J Med.

